# Spread of Human T-Lymphotropic Virus 1 and 2 Among Relatives of People Who Use Illicit Drugs in Northern Brazil

**DOI:** 10.3389/fmicb.2022.889948

**Published:** 2022-06-03

**Authors:** Aldemir Branco Oliveira-Filho, Paula Cristina Rodrigues Frade, Ricardo Roberto de Souza Fonseca, Leila Sawada, Luísa Caricio Martins, Luiz Fernando Almeida Machado, Antonio Carlos Rosário Vallinoto, Ricardo Ishak, José Alexandre Rodrigues de Lemos, Benedikt Fischer, Emil Kupek

**Affiliations:** ^1^Instituto de Estudos Costeiros, Universidade Federal do Pará, Bragança, Brazil; ^2^Programa de Pós-graduação em Doenças Tropicais, Universidade Federal do Pará, Belém, Brazil; ^3^Programa de Pós-graduação em Biologia de Agentes Infecciosos e Parasitários, Universidade Federal do Pará, Belém, Brazil; ^4^Seção de Virologia, Instituto Evandro Chagas, Ministério da Saúde, Ananindeua, Brazil; ^5^Núcleo de Medicina Tropical, Universidade Federal do Pará, Belém, Brazil; ^6^Instituto de Ciências Biológicas, Universidade Federal do Pará, Belém, Brazil; ^7^Faculty of Medical and Health Sciences, University of Auckland, Grafton, New Zealand; ^8^Centre for Applied Research in Mental Health and Addiction, Faculty of Health Sciences, Simon Fraser University, Vancouver, BC, Canada; ^9^Departamento de Saúde Pública, Universidade Federal de Santa Catarina, Florianópolis, Brazil

**Keywords:** epidemiology, HTLV, intrafamilial transmission, use of illicit drugs, prevention, public health strategies, Brazil

## Abstract

The human T-lymphotropic virus 1 (HTLV-1) and 2 (HTLV-2) can be transmitted between humans by mechanisms associated with horizontal and vertical routes. Recently, high prevalence rates and levels of genetic diversity for HTLV-1 and HTLV-2 were detected among people who use illicit drugs (PWUDs) in the Brazilian state of Pará. None of the PWUDs with HTLV-1 or HTLV-2 were aware of their carrier condition of the retrovirus, and they ability to spread it to their family group, sexual partners, and other contacts. Thus, this study evaluated the presence of HTLV-1 and HTLV-2 in families of PWUDs in the state of Pará, in Northern Brazil. This descriptive study used convenience sampling and accessed 37 PWUDs and their respective families (*n* = 97) in 18 municipalities in the state of Pará, northern Brazil. All participants provided personal data and were tested for the presence of HTLV-1 and HTLV-2 using enzyme-linked immunosorbent assay and western blotting. HTLV positive samples were selected for Nested-PCR, and viral genotyping by nucleotide sequencing and phylogenetic analysis. HTLV-1 or HTLV-2 infections were detected in 15 families of PWUDs: 27 family members of PWUDs were infected with HTLV-1 (27.8%) and another 20 of them with HTLV-2 (20.6%). Subtypes 1a [subgroup A (54.5%)], 2b (20.5%), and 2c (25.0%) were detected. High horizontal (76.9%) and vertical (61.4%) transmission rates of HTLV were ascertained. Factors that facilitate the acquisition and transmission of HTLV-1 and HTLV-2 were reported by the participants, such as long-term relationships, unprotected sex, breastfeeding, and lack of knowledge about the condition of being a carrier of the retrovirus. Evidence indicates intrafamilial transmission of HTLV from PWUDs to members of their respective families. Key interventions should urgently be employed for the control and prevention of HTLV-1 and HTLV-2 to reduce the spread of this retrovirus in PWUDs and the general population in Northern Brazil and elsewhere.

## Introduction

The human T-lymphotropic virus (HTLV) belongs to the family Retroviridae, subfamily Oncovirinae, and genus Deltaretrovirus (International Committee on Taxonomy of Viruses [ICTV], 2019). To date, four types (HTLV-1 to HTLV-4) have been described in humans, with HTLV-1 and HTLV-2 being the most prevalent (Martinez et al., 2019; Ishak et al., 2020a). There are an estimated five million individuals infected with HTLV-1 worldwide with endemic regions in Japan, Iran, Melanesia, Sub-Saharan Africa, and South America (Gessain and Cassar, 2012; González-Alcaide et al., 2016; Martinez et al., 2019). About 5% of individuals with HTLV-1 have health problems, such as tropical spastic paraparesis, leukemias, and lymphomas (Ciminale et al., 2014; Rosadas et al., 2018). On the other hand, there are an estimated 800,000 individuals infected with HTLV-2 worldwide (Martinez et al., 2019). The pathogenicity of HTLV-2 is less certain when compared to HTLV-1. There are only sporadic reports made of neurological conditions, inflammatory disorders, and leukemia possibly associated with viral infection (Martinez et al., 2019; Stufano et al., 2021). HTLV-2 is most common in members of the Pygmy tribes in Central Africa, in American indigenous groups, particularly in Amazon region, and among people who use inject drugs in the United States, Europe, and Southeast Asia (Murphy et al., 2015; Martinez et al., 2019; Ishak et al., 2020b).

Based on the nucleotide diversity of its long terminal repeat (LTR) region, there are six molecular subtypes (namely, a, b, c, d, e, and f) of HTLV-1 and four (a, b, c, and d) of HTLV-2 (Ishak et al., 2020a). Human migratory flows play an important role in the origin and spread of HTLV-1 and HTLV-2 from the African continent toward Europe, Asia, and the Americas (Ishak et al., 2020a). In South America, the original routes of infection by HTLV-1 and HTLV-2 were different and still follow different routes and modes of dispersion throughout the process of colonization and exploration of this continent (Vallinoto and Ishak, 2017; Ishak et al., 2020a). High rates of HTLV-1 and HTLV-2 infections have been recorded in Brazil, mainly in the states of Bahia, Maranhão, and Pará (Catalan-Soares et al., 2005). HTLV-1a, HTLV-2b, and HTLV-2c have shown high frequencies in studies conducted in key populations in Brazil, such as prisoners, female sex workers (FSW), people who use illicit drugs (PWUDs), and men who have sex with men (MSM) (Castro et al., 2018; Oliveira-Filho et al., 2019a; de Souza et al., 2020; Bandeira et al., 2022). Recently, two epidemiological studies conducted with PWUDs, and indigenous refugees recorded the movement of people infected with HTLV-1 (1aB) and HTLV-2 (2b and 2c) between Brazilian states and countries located in the Amazon region and evidenced the spread of these retroviruses in South America (Oliveira-Filho et al., 2019a; Abreu et al., 2022).

Interestingly, HTLV can cause silent and long-life infection in humans, alternating between persistence and production cycles, which favors efficient mechanisms for vertical and horizontal transmission, and consequently can increase the prevalence and incidence of infections and related disease outcomes (Ishak et al., 2020b). HTLV-1 and HTLV-2 can be transmitted through the transfusion of infected blood or blood products, unprotected sexual contact, sharing of contaminated syringes and other instruments, or *via* transmission from mother to child (Gonçalves et al., 2010; Paiva and Casseb, 2015; Oliveira-Filho et al., 2019a). In northern Brazil (Amazon region), there are molecular records of the spread of HTLV-1 and HTLV-2 by vertical and horizontal transmission routes in indigenous communities and in urban areas, clearly indicating the importance of these mechanisms for maintaining the endemicity of this retrovirus (Ishak et al., 2020a,b).

People who use illicit drugs represent a key population for the acquisition and spread of HTLV-1, HTLV-2, and other pathogens, especially through the sharing of paraphernalia for drug use, the timing and frequency of drug use, the type of illicit drug used, unprotected sexual activity, multiple sexual partners, and commercial sex work (Degenhardt and Hall, 2012; Oliveira-Filho et al., 2019b; Oliveira-Filho et al., 2020; Rodrigues et al., 2021). In the Brazilian state of Bahia, high prevalence rates of HTLV-1 (25.5%) and HTLV-2 (8.8%) infections have already been recorded among people who used injecting drugs. Advanced age (35–66 years) and female gender were factors associated with HTLV-1 infection, and there was no record of factors associated with HTLV-2 infection (Andrade et al., 1998). Furthermore, HTLV-2a was detected among PWUDs in Bahia, and possibly had two distinct origins: Brazilian Amerindians and European/North American PWUDs (Alcantara et al., 2003). In the Brazilian state of São Paulo, several epidemiological studies on HTLV-1 and HTLV-2 infections were conducted with people living with HIV/AIDS (PLWHAs) in recent decades. The prevalence rates of HTLV-1 (7.8–1.55%) and HTLV-2 (6.1–0.25%) have reduced in this vulnerable group, but injecting drug use has been one of the main factors associated with HTLV-1 and HTLV-2 infections (de Araujo et al., 1994; Caterino-de-Araujo et al., 1998, 2015; Jacob et al., 2008). Changing patterns of illicit drug use (from injecting cocaine to smoking crack) and harm reduction program actions (such as providing sterile syringes and needles) have been related to a reduction in the prevalence rates of these retroviruses among PLWHAs (Caterino-de-Araujo et al., 2015). Recently, high prevalence rates and levels of genetic diversity for HTLV-1 and HTLV-2 were detected among PWUDs in the Brazilian state of Pará. This fact is even more relevant because none of the PWUDs with HTLV-1 or HTLV-2 were aware of their condition as a carrier of retrovirus, and their ability to spread it to their family group, sexual partners, and other contacts (Oliveira-Filho et al., 2019a).

The intrafamilial transmission of HTLV-1 and HTLV-2 is a well-established and very important route in the epidemiological scenario. Several studies have shown the transmission of HTLV-1 and HTLV-2 through mechanisms associated with vertical (from mother to child through breastfeeding) and horizontal (unprotected sex between intimate partners) routes (Ishak et al., 2001; da Costa et al., 2013; Mendes et al., 2016; Frutos et al., 2017; Paiva et al., 2018; Rosadas and Taylor, 2019). For example, the intrafamily transmission of HTLV-1 was recorded in the metropolitan area of Belém, capital of the state of Pará: five of nine members of the same family were infected by HTLV-1, which was spread by mechanisms associated with vertical and horizontal routes, as well as two more deaths of family members from non-Hodgkin lymphoma (da Costa et al., 2013). Intrafamilial transmission may be responsible for maintaining HTLV-1 and HTLV-2 for several generations in the same family. Thus, this study evaluated the presence of HTLV-1 and HTLV-2 in families of PWUDs in the state of Pará, northern Brazil.

## Materials and Methods

### Study and Data Collection

**FIGURE 1:**
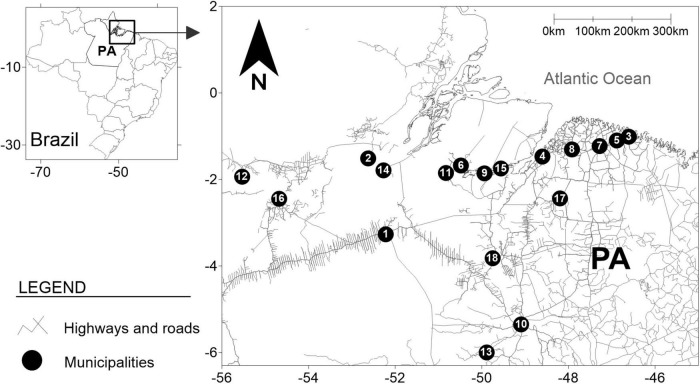
Geographic location of collection points and information on people who used illicit drugs and their families in 18 municipalities in the state of Pará, northern Brazil. Points = Municipalities: (1) Altamira (*n* = 3), (2) Almeirim (*n* = 2), (3) Augusto Corrêa (*n* = 1), (4) Belém (*n* = 3), (5) Bragança (*n* = 2), (6) Breves (*n* = 2), (7) Capanema (*n* = 2), (8) Castanhal (*n* = 2), (9) Curralinho (*n* = 3), (10) Marabá (*n* = 2), (11) Melgaço (*n* = 2), (12) Óbidos (*n* = 2), (13) Parauapebas (*n* = 1), (14) Porto de Moz (*n* = 2), (15) São Sebastião da Boa Vista (*n* = 2), (16) Santarém (*n* = 2), (17) Cametá (*n* = 2), and (18) Tucuruí (*n* = 2).

This descriptive study used convenience sampling and accessed PWUDs and their respective families in 18 municipalities in the state of Pará, northern Brazil (Figure 1). All information to access the sample of PWUDs was obtained from an epidemiological study conducted with this vulnerable group in the same Brazilian state (Oliveira-Filho et al., 2019a). Based on known HTLV-1 and HTLV-2 transmission routes, the family members of the PWUDs included were the mother; wife, husband, or similar (e.g., primary intimate partner such as girlfriend or boyfriend); and any children. Other family members (as son-in-law and daughter-in-law) were recruited based on the identification of positive cases for HTLV-1 and HTLV-2 among the personal network members already investigated. All participants provided demographic, socioeconomic, and behavioral information through a structured form, completed through a face-to-face interview. The search for PWUDs and their families and data collection was conducted from April 2019 to March 2020.

### Laboratory Diagnosis

In addition to personal (e.g., sociodemographic, behavioral) data assessed, blood samples were collected and tested for HTLV-1 and HTLV-2 infections. The presence of anti-HTLV-1/2 antibodies was evaluated by enzyme-linked immunosorbent assay (EIA) using Murex HTLV-I + II GE80/81 (DiaSorin, Italy). Non-reactive samples were classified as HTLV-negative, and no additional tests were performed on those samples. EIA-reactive samples were subjected to confirmation by Western blotting (WB) using HTLV Blot 2.4 (Genelabs Diagnostics, Singapore). All laboratory tests were conducted according to manufacturers’ guidelines.

### Sequence and Phylogenetic Analysis

All samples positive for anti-HTLV-1/2 antibodies by EIA and WB were selected for the isolation of nucleic acid using the PureLinkTM Viral RNA/DNA Mini Kit (Invitrogen, Carlsbad, CA, United States), and then subjected to Nested-PCR for fragment amplification of the 5′ LTR (de Oliveira et al., 2012). The PCR products were subjected to 1.5% agarose gel electrophoresis and subsequently purified using QIAquick PCR Purification Kit (Qiagen, Valencia, CA, United States). The nucleotide sequencing was performed using the BigDyeTerminator 3.1 kit (Applied Biosystems, Foster City, CA, United States) by capillary electrophoresis on the ABI PRISM 3130 system (Applied Biosystems, Foster City, CA, United States).

All sequences were edited and aligned using the BioEdit^1^ and AliView (Larsson, 2014) programs, respectively. The subtypes were determined using phylogenetic analyses with HTLV reference sequences (Supplementary Table 1), obtained from the National Center for Biotechnology Information.^2^ To verify the clustering of HTLV sequences, maximum-likelihood (ML) phylogenetic trees were reconstructed with the PhyML 3.1 (Guindon et al., 2010) under the best nucleotide substitution model, selected by the Smart Model Selection (SMS) software integrated into the PhyML Web server (Lefort et al., 2017). The heuristic trees search was performed using the SPR branch-swapping algorithm, and the branch support was calculated with the approximate likelihood-ratio (aLRT) Shimodaira–Hasegawa test (Supplementary Table 2). The tree was drawn with FigTree 1.4.4.^3^ The sequences obtained in this study were deposited in GenBank (OM835810-OM835889).

### Statistical Analysis

In this study, a set of family members was generated from each of the PWUDs. HTLV-1 or HTLV-2 infection was defined by the presence of antibodies using EIA and WB. The horizontal (e.g., through sexual partners: spouse-to-spouse or similar) and vertical (e.g., mother-to-child) transmission routes for HTLV-1 and HTLV-2 were considered and highlighted (when members of the same family were infected with the same HTLV-1 or HTLV-2 subtype; Supplementary Figure 1). The collected data were organized and described in absolute and relative frequencies.

## Results

**FIGURE 2:**
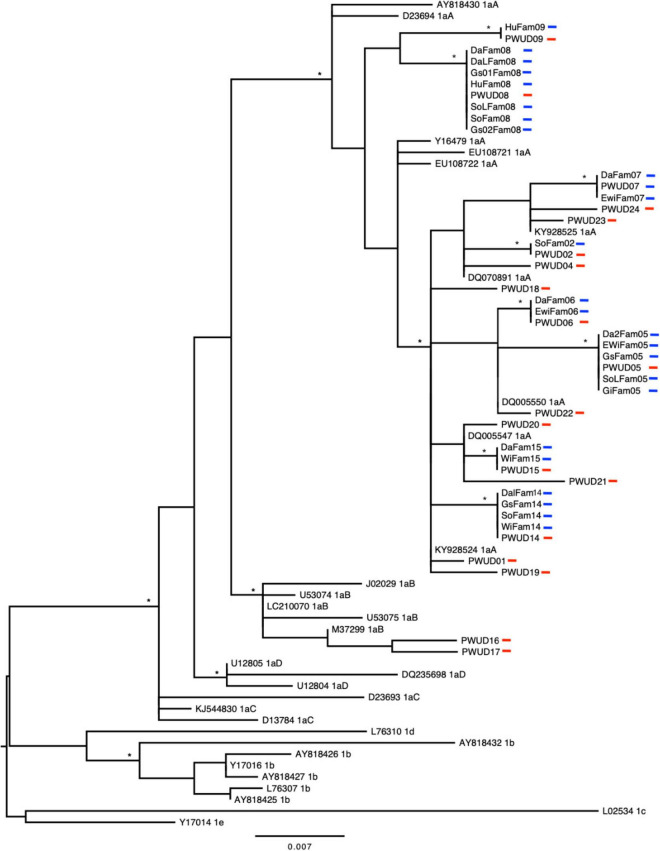
Phylogenetic tree constructed by maximum likelihood using long terminal repeat sequences (421 base pairs) belonging to the HTLV-1 detected in people who use illicit drugs (PWUDs) and their families in the Brazilian state of Pará, and other sequences obtained from GenBank. The tree was rooted at the midpoint. Asterisks point to key nodes with high support (aLRT ≥ 0.95). The samples in this study can be identified by traces in red (PWUDs) and blue (their relatives).

**FIGURE 3:**
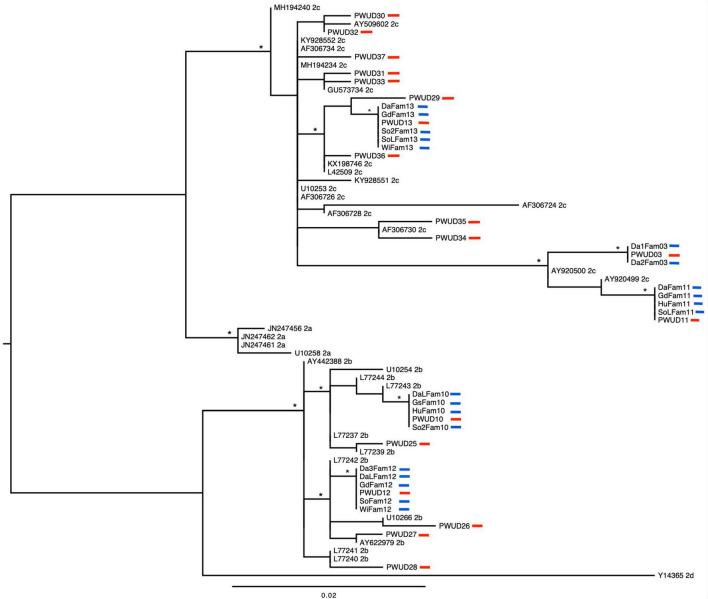
Phylogenetic tree constructed by maximum likelihood using long terminal repeat sequences (388 base pairs) belonging to the HTLV-2 detected in people who use illicit drugs (PWUDs) and their families in the Brazilian state of Pará, and other sequences obtained from GenBank. The tree was rooted at the midpoint. Asterisks point to key nodes with high support (aLRT ≥ 0.95). The samples in this study can be identified by traces in red (PWUDs) and blue (their relatives).

Initially, 53 PWUDs with positive results for anti-HTLV-1/2 antibodies were searched, but only 37 were found (nine PWUDs had died, and the other seven were not found in their home locales) and participated in this study. In this sample (*n* = 37), the majority were men, single, with up to 10 years of education, and low monthly income (Table 1). The mean age was 39.6 years (±6.5). All PWUDs reported having used more than one illicit drug in their lifetime and frequent use of non-injecting drugs in the last 12 months, such as crack and cocaine. Several behaviors that facilitate the acquisition and transmission of HTLV-1 and HTLV-2 were reported by PWUDs, such as unprotected sex, multiple sexual partners, exchanging sex for money or drugs, and sharing of drug use paraphernalia (Table 1). All PWUDs were tested positive for anti-HTLV antibodies using EIA and WB: 51.4% were HTLV-1 and 48.6% were HTLV-2 positive (Table 2). Subtypes 1a [51.4%; subgroups 1aA (46.0%) and 1aB (5.4%)], 2b (16.2%) and 2c (32.4%) were detected (Figures 2, 3 and Supplementary Tables 1, 2). All PWUDs were aware of their HTLV-1 or HTLV-2 carrier status because of participating in a scientific study in the state of Pará, and most of them still repeated the same risk behaviors for viral infections (Table 1). However, only five of them sought medical care related to viral infections but abandoned such care due to the financial costs related to travel for medical consultations and laboratory tests. They lived in municipalities far (Altamira, Almeirim, Melgaço, Óbidos, and Porto de Moz) from the large metropolitan area of Belém, where specialized care for HTLV-1 or HTLV-2 infections available in the state of Pará.

**TABLE 1:** Key characteristics or behaviors of people who use illicit drugs in the Brazilian state of Pará.

Characteristics/behaviors	*n* (%)
**Gender**	
Male	29 (78.4)
Female	8 (21.6)
**Age**	
18–29 years	9 (24.3)
30–39 years	20 (54.1)
40+ years	8 (21.6)
**Sexual orientation**	
Heterosexual	37 (100.0)
Marital status*	
Single	29 (78.4)
Married or co-habitating	8 (21.6)
**Education**	
Up to 10 years of study	21 (56.8)
More than 10 years of study	16 (43.2)
Monthly income*	
≤1 minimum wage**	24 (64.8)
2–3 times minimum wage	13 (35.2)
Main illicit drug used*	
Crack	22 (59.5)
Cocaine (powder or paste)	15 (40.5)
**Period of illicit drug use**	
Up to 10 years	16 (43.2)
More than 10 years	21 (56.8)
Use of injectable cocaine***	
Yes	15 (40.5)
No	22 (59.5)
Sharing of drug use paraphernalia*	
Yes	28 (75.7)
No	9 (24.3)
Unprotected sex (vaginal and/or anal)*	
Yes	34 (91.9)
No	3 (8.1)
Number of sexual partners*	
Up to five	14 (37.8)
More than five	23 (62.2)
Exchanging sex for money or drugs*	
Yes	22 (59.5)
No	15 (40.5)

*Last 12 months; **Average of Brazilian minimum wage equals 1073 Reais (equivalent to 215 US dollars) from 2019 to 2020; ***At least once in your life.

**TABLE 2:** Presence and possible transmission routes of HTLV-1 and HTLV-2 in people who use illicit drugs (PWUDs) and their families in the state of Pará, northern Brazil.

Study participants	HTLV+/Total (%)	HTLV-1/Total	HTLV-2/Total (%)
PWUDs	37/37 (100.0)	19/37 (51.4)	18/37 (48.6)
Families of PWUDs	15/37 (40.5)	10/37 (27.0)	5/37 (13.5)
PWUD family members	47/97 (48.4)	27/97 (27.8)	20/97 (20.6)
Mother	0/27 (0.0)	0/27 (0.0)	0/27 (0.0)
Husband, wife, ex-wife, or girlfriend	12/16 (75.0)	8 (50.0)	4 (25.0)
Son or Daughter	19/31 (61.3)	11 (35.5)	8 (25.8)
Grandson or granddaughter	8/13 (61.6)	4 (30.8)	4 (30.8)
Son-in-law or daughter-in-law	8/10 (80.0)	4 (40.0)	4 (40.0)
**Possible route of viral transmission within families**			
Horizontal (unprotected sex)	20/26 (76.9)	12/26 (46.2)	8/26 (30.7)
Vertical (breastfeeding)	27/44 (61.4)	15/44 (34.1)	12/44 (27.3)

In addition, 37 families of PWUDs were evaluated. Most families (*n* = 18) presented as members the person who used illicit drugs and his mother, without any indication of a steady sexual partner or children. In total, 119 family members of PWUDs were identified, but only 97 of them were found and participated in the study as described here: mother (*n* = 27), husband (*n* = 4), wife (*n* = 4), steady sexual partner (girlfriend) (*n* = 5), ex-wife (*n* = 3), son (*n* = 15), daughter (*n* = 16), grandson (*n* = 11), granddaughter (*n* = 2), daughter-in-law (*n* = 5), and son-in-law (*n* = 5). Ten mothers and two ex-wives of PWUDs had died, and two ex-wives, five daughters, and three sons were not found in the PWUDs’ home municipality. In the sample of family members of PWUDs (*n* = 97), most were female (63.9%), had up to 10 years of education (72.2%), and had low monthly income (83.5%). The family members’ ages ranged from 1 to 81 years. None of these family members reported: use of illicit drugs in life, exchange of sex for money or drugs during life, and more than one sexual partner in the last 12 months. All PWUDs husbands, wives, and steady sexual partners reported unprotected sex in the last 12 months. PWUDs’ ex-wives haven’t had sex with them for over 24 months.

Human T-lymphotropic virus 1 or human T-lymphotropic virus 2 infections were detected in 15 families of PWUDs (Table 2 and Supplementary Figure 1). In total (*n* = 97), 27 family members of PWUDs were infected with HTLV-1 (27.8%) and another 20 of them with HTLV-2 (20.6%) (Table 2). No cases of co-infection were detected. Proviral DNA was detected in most samples from family members with HTLV-1 or HTLV-2 infection (*n* = 44). No proviral DNA has been detected in three samples (two sons and a daughter of PWUDs) that tested positive for HTLV-1 using EIA and WB. Subtypes 1a [subgroup A or Transcontinental (54.5%)], 2b (20.5%) and 2c (25.0%) were detected in the proviral DNA samples (Figures 2, 3 and Supplementary Tables 1, 2). In Figure 2, eight well-defined clusters (nodes with high support, aLRT ≥ 0.95), belonging to subgroup 1aA, stand out for their composition. All are composed of PWUDs and their relatives. In Figure 3, a similar case can also be seen, two clusters belonging to subtype 2b and three other clusters belonging to subtype 2c were detected, all well-defined (nodes with high support, aLRT ≥ 0.95), and composed of PWUDs and their respective relatives.

Most family members of PWUDs with HTLV-1 or HTLV-2 infection were female (53.2%) and had up to 10 years of education (66.0%) or were illiterate (25.5%). The age ranged from 1 to 44 years (average age = 21.7 years). All mothers of the PWUDs tested negative for HTLV-1 or HTLV-2. Husbands (*n* = 4), wives (*n* = 4), ex-wives (*n* = 3), girlfriend (*n* = 1), daughters-in-law (*n* = 4), and sons-in-law (*n* = 4) were detected with HTLV-1 or HTLV-2, and evidenced the possibility of horizontal transmission, as they reported unprotected sex with infected sexual partners (Table 2). The duration of the intimate relationship between these sexual partners ranged from 6 months to 25 years. Four sexual partners (girlfriends) of PWUDs tested negative for HTLV, they had been in a relationship for 2–3 months. On the other hand, the detection of HTLV-1 or HTLV-2 among sons (*n* = 8), daughters (*n* = 11), grandson (*n* = 6), and granddaughters (*n* = 2) of PWUDs evidenced the possibility of vertical transmission through breastfeeding from infected mother to child (Table 2). All sons, daughters, grandsons, and granddaughters with HTLV-1 or HTLV-2 were breastfed only by their respective mothers, who tested positive for HTLV-1 or HTLV-2.

**FIGURE 4:**
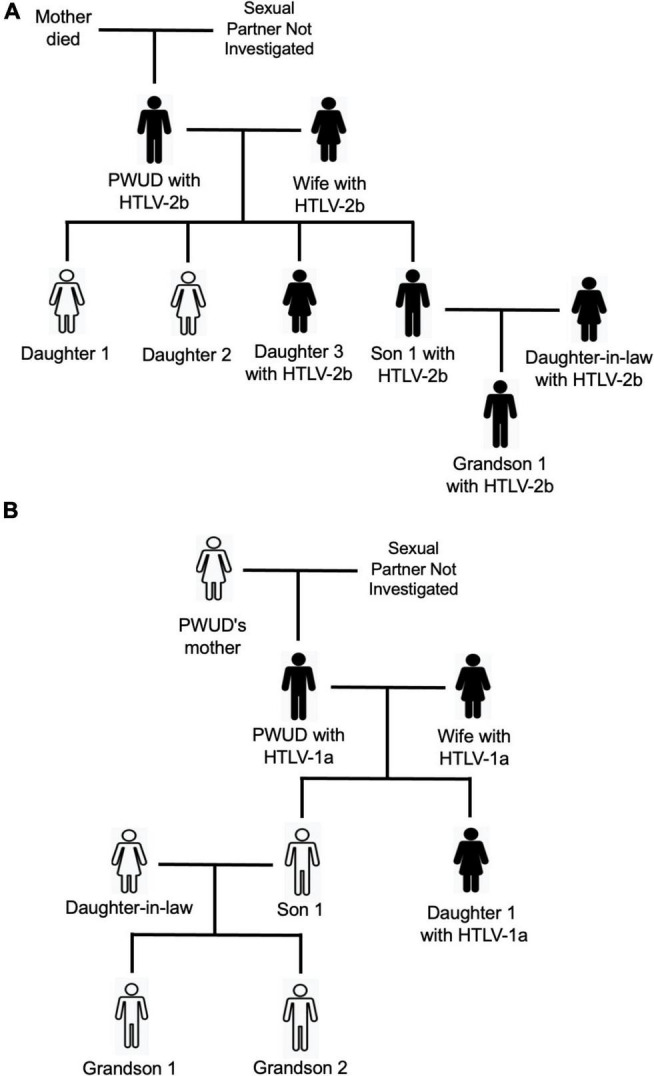
Schematic representations of the spread of HTLV-1 and HTLV-2 in families of people who use illicit drugs (PWUDs) in the state of Pará, northern Brazil. **(A)** Family with members infected by HTLV-2. **(B)** Family with members infected with HTLV-1a.

The schematic representations of the PWUD families allowed a clearer view of the entry and spread of HTLV-1 or HTLV-2 in these groups. For example, in Figure 4A, the PWUD’s wife was infected with HTLV-2b, possibly through unprotected sex. Daughter 3 and Son 1 were generated after the mother’s infection and were infected with the same viral subtype through breastfeeding. Thereafter, HTLV-2b was transmitted from Son 1 (unaware of HTLV-2b carrier status) to his wife through unprotected sex, who transmitted this retrovirus to her child (Grandson 1) through breastfeeding. In Figure 4B, it is also possible to identify the possibility of HTLV-1a transmission *via* horizontal (PWUD’s wife infected by HTLV-1a through unprotected sex) and vertical (daughter 1 infected by HTLV-1a through breastfeeding) routes. All family members of PWUDs were unaware that they were infected with HTLV-1 or HTLV-2, and there were no reports of symptoms related to these viral infections. Other schematic representations indicate the spread of HTLV-1 and HTLV-2 in families of the PWUDs in Supplementary Figure 1, including HTLV-1a (families 2, 6, and 9) and HTLV-2b (family 12) in families of people who have used injectable cocaine at least once in their lifetime.

## Discussion

This study recorded the presence of HTLV-1 and HTLV-2 among PWUDs and in several members of their families. A high positivity rate of HTLV infection was detected among sexual partners of PWUDs (76.9%), as well as sexual partners of sons and daughters of PWUDs, indicating the importance of the horizontal route for the spread of this virus in the general population. Similarly, a high rate of positivity of HTLV infection was detected in sons, daughters, grandsons, and granddaughters of PWUDs (61.4%), clearly indicating the silent but substantive contribution of the vertical route to the maintenance of high rates of this viral infection, such as prevalence and incidence, by generations. These values are higher than what has been recorded in an epidemiological investigation on mother-child (20.0–21.2%) and spouse-spouse (14.3–51.3%) dyads of patients with HTLV-1 and HTLV-2 treated at the referral center in the metropolitan area of Belém (da Costa et al., 2013). Furthermore, viral subtypes and subgroups detected here have already been reported in other epidemiological studies conducted with different population groups in northern Brazil (Laurentino et al., 2005; Vallinoto et al., 2006; Santos et al., 2009; de Aguiar et al., 2017; Braço et al., 2019; Oliveira-Filho et al., 2019a; de Souza et al., 2020; Ishak et al., 2020b), other Brazilian regions (Renner et al., 2006; Novoa et al., 2007; de Almeida Rego et al., 2010; de Oliveira et al., 2012; Magri et al., 2012; Pessôa et al., 2014; Bandeira et al., 2015, 2022; Castro et al., 2018; Ribeiro et al., 2018), and in South American countries (Leon-Ponte et al., 1996; Miura et al., 1997; Talarmin et al., 1999; Berini et al., 2013; Abreu et al., 2022). However, the same viral subtype detected in 15 PWUDs was detected in their family members. Thus, there is evidence of the spread of HTLV-1 and HTLV-2 through intrafamilial transmission, by procedures associated with the horizontal route as well as the vertical route.

In general, the acquisition and spread of HTLV-1 and HTLV-2 among PWUDs occur mainly through risky sexual contact, and this can be potentiated by different patterns and forms of illicit drug use (Oliveira-Filho et al., 2019a). The long-term relationship and the occurrence of unprotected sex between PWUDs and their sexual partners are relevant factors for the introduction and spread of HTLV-1 or HTLV-2 within families. Following a couple’s viral infection, this retrovirus can be easily spread to the next generation of the family (sons and daughters) as facilitated by two other main factors, namely, the lack of knowledge of the carrier status of the virus and breastfeeding. The couple’s offspring may also spread HTLV-1 or HTLV-2 through unprotected sex to their sexual partner (daughters-in-law and sons-in-law), and sons and daughters from this relationship (grandchildren and granddaughters) through, again, the lack of knowledge of the retrovirus carrier status and breastfeeding. Likely, these pathways of introduction and spread of HTLV has occurred in several families evaluated in this study. There are several scientific records showing the importance of these factors for the spread of HTLV-1 and HTLV-2, mainly in small groups as families (Houinato et al., 1998; Ishak et al., 2001; da Costa et al., 2013; Frutos et al., 2017; Bandeira et al., 2018; Ribeiro et al., 2018).

This finding is very important for understanding the epidemiological scenario of HTLV-1 and HTLV-2 in northern Brazil (Amazon region), as it indicates how this retrovirus has been transmitted “silently” for generations. Consequently, there are key interventions to be highlighted for the control and prevention of HTLV-1 and HTLV-2 in this Brazilian region. First, there is a need to provide expanded laboratory testing and surveillance for HTLV-1 and HTLV-2, and clinical guidance and supervision of people living with this retrovirus, especially in municipalities far from the state of Pará’s capital. The concentration of resources, services, and skills techniques exclusively in the metropolitan area of Belém complicates the diagnosis and clinical care of people infected with HTLV-1 and HTLV-2 living in more distant municipalities, such as Almeirim, Óbidos, Melgaço and, Porto de Moz, or rural areas of the state. The precarious transport network and the deficiency of health services in this Brazilian region associated with low education and low economic income of most of the population are obstacles that must be considered and addressed toward generating desirable changes and improvements in the present epidemiological scenario. One suggestion is the establishment of advanced units for diagnosis, guidance, and clinical care of people with HTLV-1, HTLV-2, and other pathogens in microregions within the immense state of Pará. Access to diagnosis is an essential tool for surveillance, prevention, and clinical monitoring, especially in an area with a high rate of infection (Oliveira-Filho et al., 2019a).

Another key intervention is the need for better primary prevention of HTLV infections. Unprotected sex and breastfeeding are important primary risk factors for the spread of this retrovirus. Therefore, education programs aimed at reducing risky sexual behavior must be implemented by public health authorities, including the distribution of resources or prevention materials such as male and female condoms. Yet, the need to better integrate and address HTLV in campaigns for the control and prevention of STIs in the general population should be highlighted. This becomes even more relevant because of the easy viral acquisition and spread in key populations such as PWUDs, who even knowing their HTLV carrier status still engage in unprotected sex and other risky behaviors. The sub-population of pregnant women should be monitored more carefully. Recently, a flowchart for the management of HTLV infection in pregnant women and prevention of mother-to-child transmission has been proposed and showed well-defined measures for monitoring, prevention, and guidance of mothers and neonates in the prenatal, delivery, and post-partum phases (Rosadas et al., 2021). In general, these health strategies can prevent the transmission of HTLV from mother to child or between sexual partners, and their widespread implementation in the state of Pará and other states in northern Brazil is essential.

Considering the duration and intensity of illicit drug use reported by PWUDs in this study, another key intervention is the treatment of drug problems in this particular risk population, including the availability of resources to minimize the harm associated with the use of psychoactive substances. This could reduce the spread of HTLV and other pathogens in the general population, as well as provide better life expectancy and quality of life for PWUDs. Most participants are likely to need treatment for their drug use, which in many instances includes one or multiple drug use disorders (e.g., dependence) (Vergara-Moragues et al., 2014). Mental comorbidities are also disproportionately common in people who regularly use illicit drugs, and further amplify the risk for virus transmission through behavioral risk factors (Narvaez et al., 2014; Vergara-Moragues et al., 2014). This study overall reinforces the urgent need to improve the availability and access to diagnosis, prevention, and treatment of PWUDs in northern Brazil.

This study has limitations and should be highlighted. It has a small number of participants, because of the difficulty in accessing the PWUDs, and consequently their families, and the complex logistics for the movement of people and products in the Brazilian state of Pará (Amazon region). The descriptive nature of the study is also a limiting factor. Evidence of intrafamilial transmission of HTLV-1 and HTLV-2 is supported only by phylogenetic findings. Other information and resources could have been accessed and used to support or exclude the possibility of intrafamilial transmission of these retroviruses. A complex suggestion to minimize all these problems is to conduct a prospective study, with a larger sample size, using robust epidemiological methods and accurate laboratory diagnoses for the identification of HTLV-1 and HTLV-2 and, consequently, a more accurate assessment of the possibility of intrafamilial transmission.

In summary, this study detected the presence of HTLV-1 and HTLV-2 in PWUDs and several members of their families, as well as documented high rates of transmission of this retrovirus through horizontal (e.g., unprotected sex) and vertical (e.g., breastfeeding) routes. Evidence indicates intrafamilial transmission of HTLV subtypes 1a, 2b, and 2c of PWUDs to their respective relatives. Key interventions should urgently be employed for the improved control and prevention of HTLV-1 and HTLV-2 in the state of Pará to reduce the spread of this retrovirus in PWUDs and the general population, represented here by PWUD family members.

## Data Availability Statement

The datasets presented in this study can be found in online repositories. The names of the repository/repositories and accession number(s) can be found below: GenBank (OM835810-OM835889).

## Ethics Statement

The studies involving human participants were reviewed and approved by Ethics Committee on Research Involving Human Subjects of the Federal University of Pará, Brazil (CAAE: 37536314.4.0000.5172). The patients/participants provided their written informed consent to participate in this study.

## Author Contributions

AO-F, LCM, JR, BF, and EK: conceptualization. PF, RF, LS, LCM, LFAM, JR, AV, and RI: investigation and methodology. AO-F, PF, and RF: formal analysis. AO-F: writing–original draft and project administration. PF, RF, LS, LFAM, AV, RI, BF, and EK: writing–review and editing. AO-F, LCM, JR, and BF: funding acquisition. All authors contributed to the development of research, contributed to the article, and approved the submitted version.

## Conflict of Interest

The authors declare that the research was conducted in the absence of any commercial or financial relationships that could be construed as a potential conflict of interest.

## Publisher’s Note

All claims expressed in this article are solely those of the authors and do not necessarily represent those of their affiliated organizations, or those of the publisher, the editors and the reviewers. Any product that may be evaluated in this article, or claim that may be made by its manufacturer, is not guaranteed or endorsed by the publisher.

## Footnotes

1
https://bioedit.software.informer.com

2
https://www.ncbi.nlm.nih.gov

3
http://tree.bio.ed.ac.uk/software/figtree
