# Supplementary material for: Spread of Human T-Lymphotropic Virus 1 and 2 Among Relatives of People Who Use Illicit Drugs in Northern Brazil
Source: Front Microbiol. 2022 Jun 3;13:889948. doi: 10.3389/fmicb.2022.889948 (PMC9205188; doi:10.3389/fmicb.2022.889948)
Supplement: Supplementary file 1 [file Data_Sheet_1.PDF]

*Supplementary Material*

**Table S1:** HTLV-1 and HTLV-2 nucleotide sequences deposited in GenBank and used as references in this study.

| Access number | Types and subgroups | Isolate | Geographic origin |
|---|---|---|---|
| DQ070891 | 1aA | SNT43 | Brazil |
| KY928524 | 1aA | BRSP71066-15 | Brazil |
| DQ005547 | 1aA | FNN100 | Brazil |
| DQ005550 | 1aA | FNN153 | Brazil |
| Y16475 | 1aA | Qu1_Peru | Peru |
| Y16479 | 1aA | Me2_Peru | Peru |
| AY818430 | 1aA | 2472LE | Cameroon |
| D23694 | 1aA | Ainu | Japan |
| KY928524 | 1aA | CA423 | Brazil |
| KY928525 | 1aA | BRSP87092-14 | Brazil |
| EU108722 | 1aA | CA253 | Brazil |
| EU108721 | 1aA | CA422 | Brazil |
| U53074 | 1aB | TA6 | Taiwan |
| U53075 | 1aB | TA7 | Taiwan |
| LC210070 | 1aB | JPNBr177 | Japan |
| J02029 | 1aB | ATK | Japan |
| M37299 | 1aB | H5 | Japan |
| KJ544830 | 1aC | PH906 | Senegal |
| D23693 | 1aC | GH78 | Japan |
| D13784 | 1aC | HS35 | Caribbean islands |
| U12804 | 1aD | Bo | Algeria |
| U12805 | 1aD | OD | Mauritania |
| DQ235698 | 1aD | BD89112 | Senegal |
| AY818427 | 1b | 1503MV | Cameroon |
| AY026855 | 1b | 2656ND | Central Africa |
| AY818423 | 1b | 979MO | Cameroon |
| AY818425 | 1b | 1380MV | Cameroon |
| AY818426 | 1b | 1443MV | Cameroon |
| AY818429 | 1b | 1842LE | Cameroon |
| AY818432 | 1b | 2810YI | Cameroon |
| AY818433 | 1b | 1127MO | Cameroon |
| AY818424 | 1b | 1259NG | Cameroon |

| | | | |
|---|---|---|---|
| Y17016 | 1b | Lib1 | Gabon |
| Y17017 | 1b | Lib2 | Gabon |
| L76307 | 1b | PH236 | Gabon |
| L76306 | 1b | SDen | Gabon |
| Z32527 | 1b | ITIS | Congo/Zaire |
| L76312 | 1b | H23 | Cameroon |
| L02534 | 1c | Me15 | Melanesia |
| L76310 | 1d | PYG19 | Central African Republic |
| Y17014 | 1e | Efe1 | Congo/Zaire |
| U10258 | 2a | NOR2N | Norway |
| JN247462 | 2a | LA8A | Indonesia |
| JN247456 | 2a | 09IDSKAH-1-2 | Indonesia |
| JN247461 | 2a | PUEB_2 | Indonesia |
| Y14365 | 2d | Pygmy2 | Congo/Zaire |
| U10266 | 2b | SPAN130 | Spain |
| U10254 | 2b | ITA47A | Italy |
| AY442388 | 2b | SP25 | Spain |
| L77237 | 2b | DP | Southern Europe |
| L77242 | 2b | 130 | Southern Europe |
| L77240 | 2b | JL | Southern Europe |
| L77243 | 2b | 324 | Southern Europe |
| L77244 | 2b | RVP | Southern Europe |
| L77239 | 2b | JA | Southern Europe |
| L77241 | 2b | JAN | Southern Europe |
| AY622979 | 2b | PortVs | Portugal |
| AY509602 | 2c | BH339 | Brazil |
| MH194240 | 2c | BrPa_Kat116 | Brazil |
| MH194234 | 2c | BrPa_Dju214 | Brazil |
| U10253 | 2c | BRAZ.A21 | Brazil |
| L42509 | 2c | KAY73 | Brazil |
| AF306730 | 2c | TYR8680 | Brazil |
| AF306728 | 2c | GTY9274 | Brazil |
| AF306734 | 2c | BBD4410 | Brazil |
| KX198746 | 2c | PI46 | Brazil |
| AF306724 | 2c | Bel3948 | Brazil |
| AY920499 | 2c | Bel-11935 | Brazil |
| AY920500 | 2c | Bel-11507 | Brazil |
| GU573734 | 2c | Bel-10562 | Brazil |
| KY928552 | 2c | BRSP96617-14 | Brazil |
| KY928551 | 2c | BRSP92116-1 | Brazil |
| AF306726 | 2c | BEL9083 | Brazil |

**Table S2:** Evolutionary models and other information used to reconstruct phylogenetic trees and identify HTLV-1 and HTLV-2 subtypes and subgroups in this study.

| | |
|---|---|
| **HTLV-1:** | |
| Model of nucleotides substitution | HKY85 |
| Log-likelihood | -1429.86578 |
| Discrete gamma model | Yes |
| Gamma shape parameter | 0.149 |
| Transition/transversion ratio | 10.906310 |
| **HTLV-2:** | |
| Model of nucleotides substitution | GTR |
| Log-likelihood | -1176.16034 |
| Discrete gamma model | Yes |
| Gamma shape parameter | 0.253 |
| GTR relative rate parameters | A <-> C: 0.71638<br>A <-> G: 5.71992<br>A <-> T: 0.05875<br>C <-> G: 0.80443<br>C <-> T: 3.64217<br>G <-> T: 1.00000 |

**Figure S1:** Schematic representation of 15 families that have a person who used illicit drugs (PWUD) with HTLV-1/HTLV-2 infection in northern Brazil.

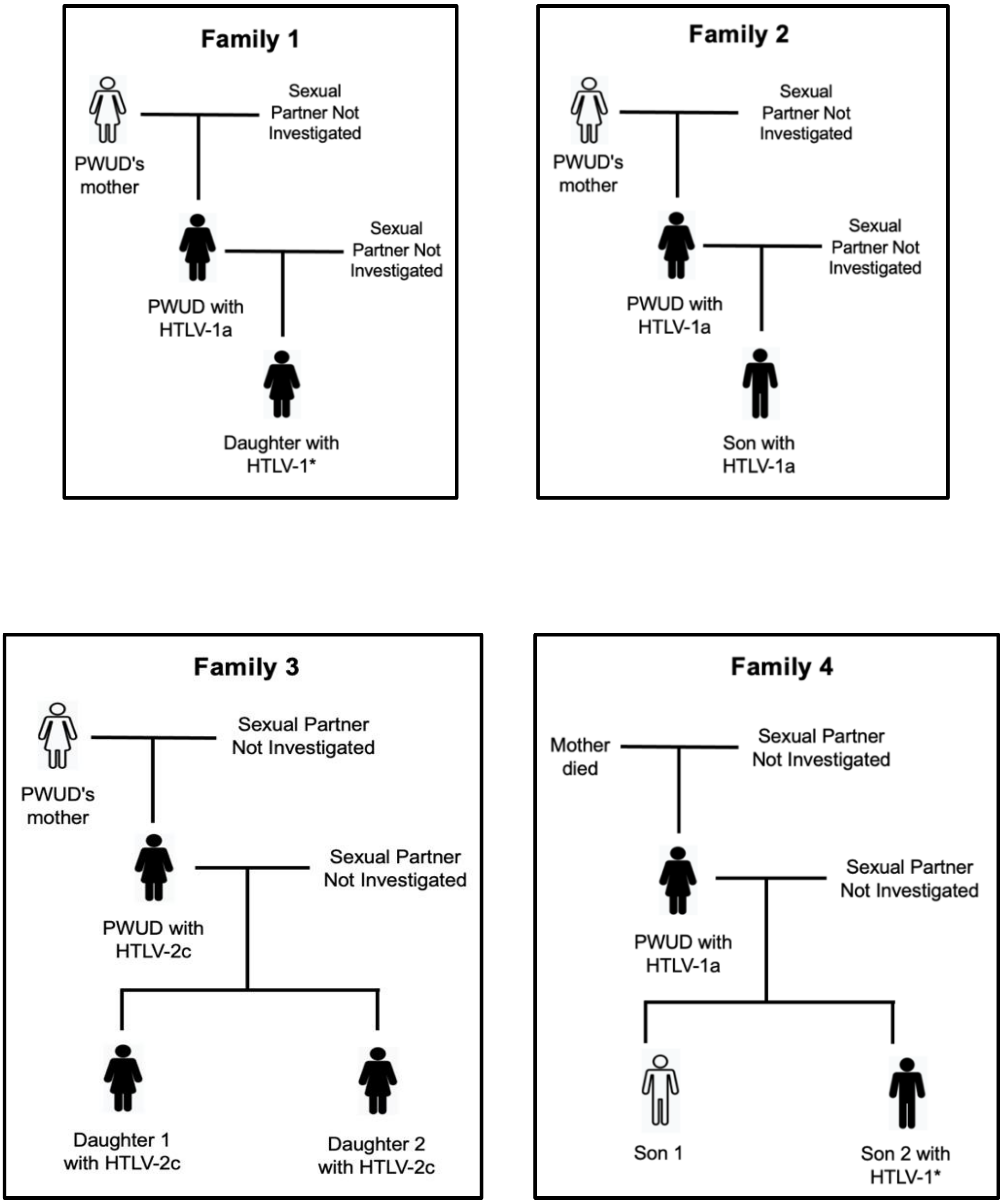

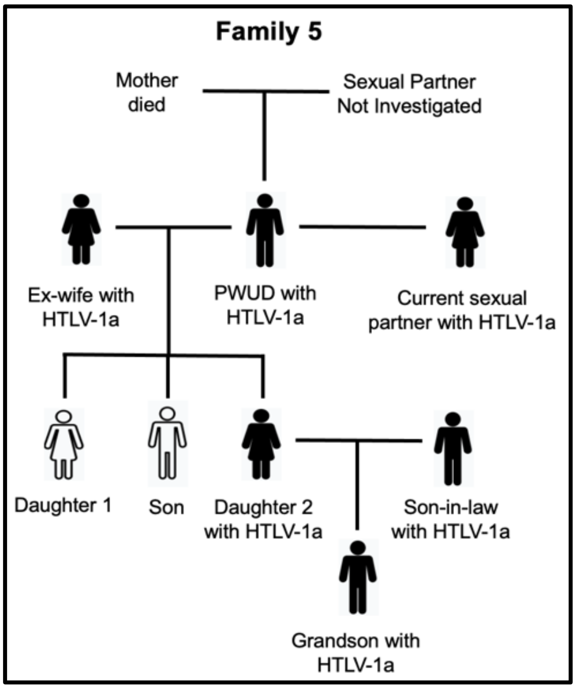
Family 5
Mother died
Sexual Partner Not Investigated
Ex-wife with HTLV-1a
PWUD with HTLV-1a
Current sexual partner with HTLV-1a
Daughter 1
Son
Daughter 2 with HTLV-1a
Son-in-law with HTLV-1a
Grandson with HTLV-1a

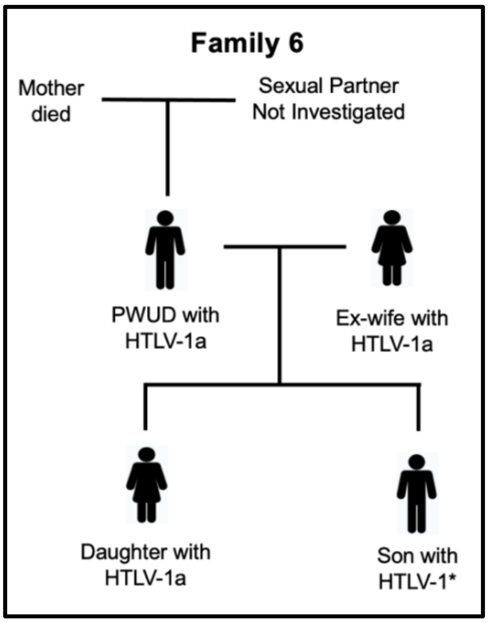
Family 6
Mother died
Sexual Partner Not Investigated
PWUD with HTLV-1a
Ex-wife with HTLV-1a
Daughter with HTLV-1a
Son with HTLV-1*

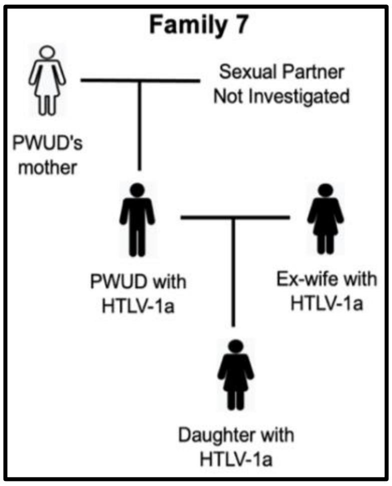
Family 7
PWUD's mother
Sexual Partner Not Investigated
PWUD with HTLV-1a
Ex-wife with HTLV-1a
Daughter with HTLV-1a

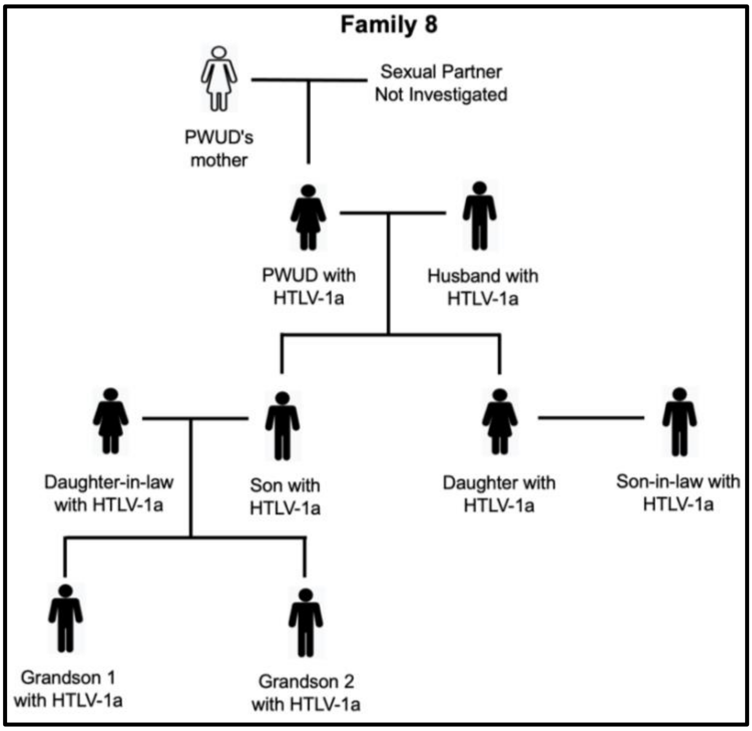
Family 8
PWUD's mother
Sexual Partner Not Investigated
PWUD with HTLV-1a
Husband with HTLV-1a
Daughter-in-law with HTLV-1a
Son with HTLV-1a
Daughter with HTLV-1a
Son-in-law with HTLV-1a
Grandson 1 with HTLV-1a
Grandson 2 with HTLV-1a

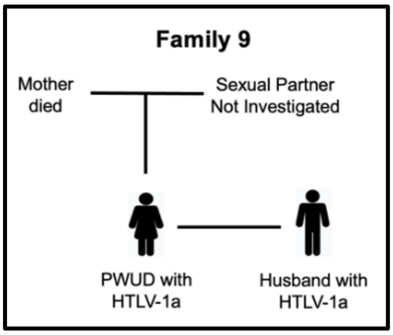
Family 9
Mother died
Sexual Partner Not Investigated
PWUD with HTLV-1a
Husband with HTLV-1a

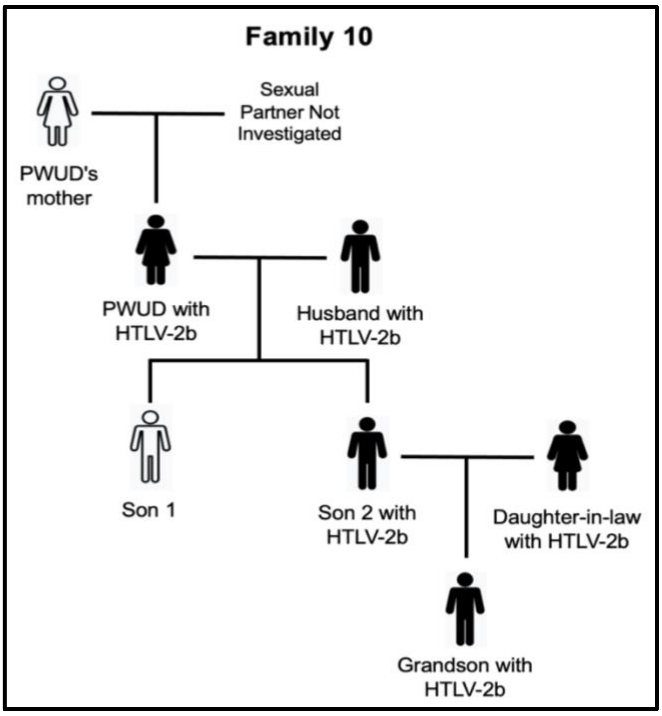
Family 10
Sexual Partner Not Investigated
PWUD's mother
PWUD with HTLV-2b
Husband with HTLV-2b
Son 1
Son 2 with HTLV-2b
Daughter-in-law with HTLV-2b
Grandson with HTLV-2b

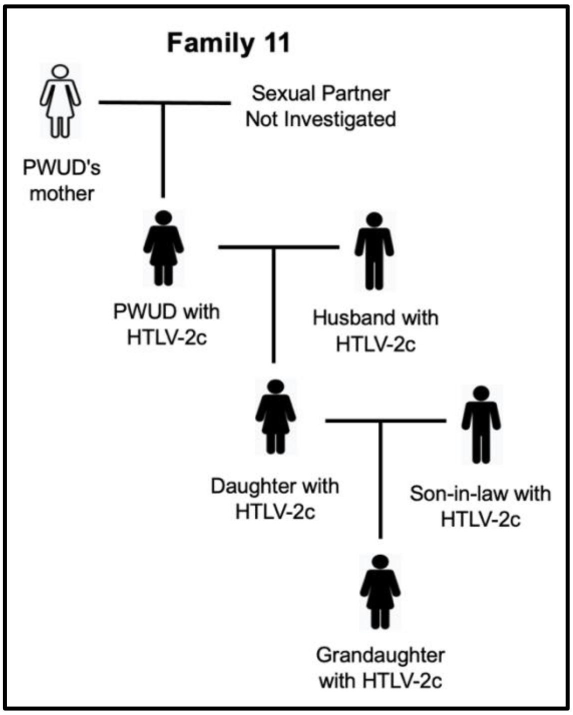
Family 11
Sexual Partner Not Investigated
PWUD's mother
PWUD with HTLV-2c
Husband with HTLV-2c
Daughter with HTLV-2c
Son-in-law with HTLV-2c
Grandaughter with HTLV-2c

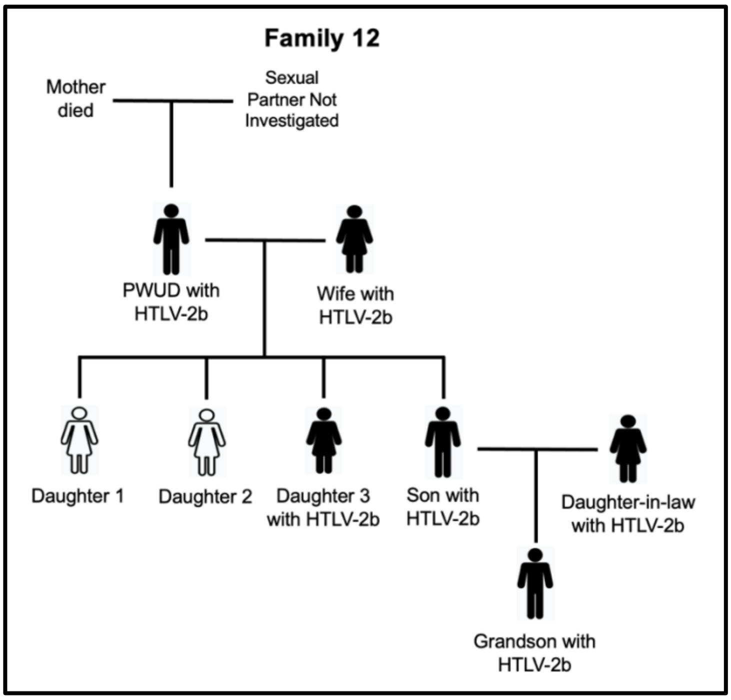
Family 12
Mother died
Sexual Partner Not Investigated
PWUD with HTLV-2b
Wife with HTLV-2b
Daughter 1
Daughter 2
Daughter 3 with HTLV-2b
Son with HTLV-2b
Daughter-in-law with HTLV-2b
Grandson with HTLV-2b

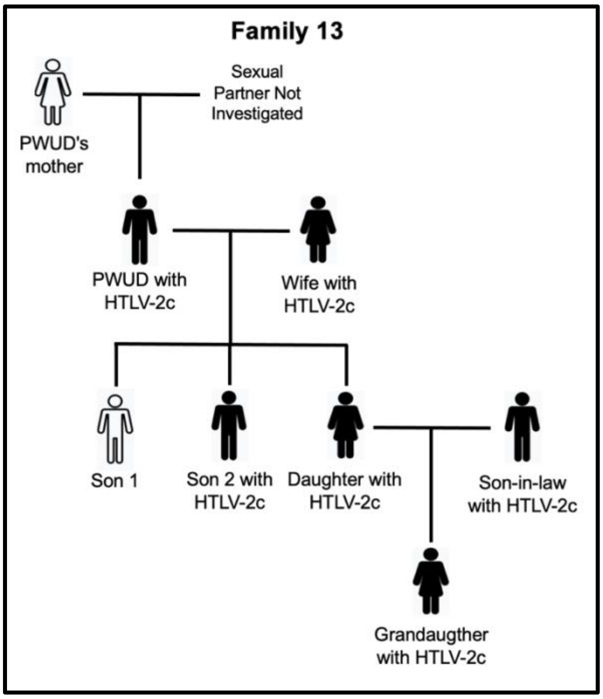


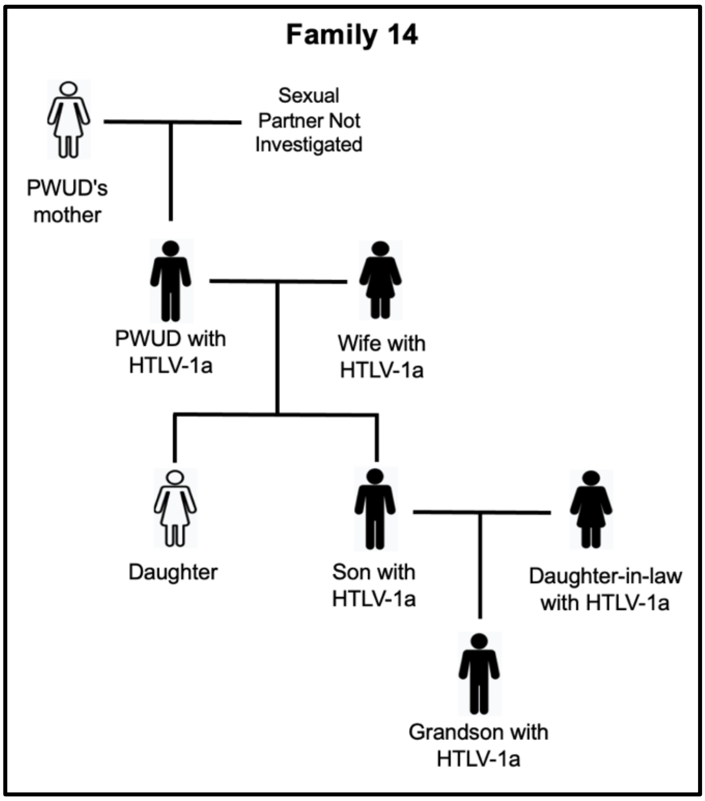


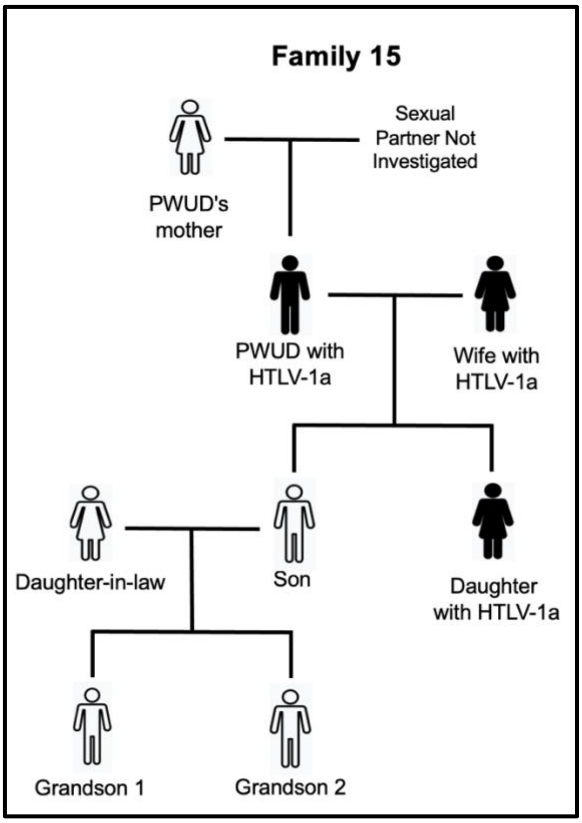


* Proviral DNA was not detected in this participant's sample.
